# No strategic interactions between sequential grasping actions

**DOI:** 10.3389/fnhum.2026.1870665

**Published:** 2026-06-25

**Authors:** Kevin A. LeBlanc, David A. Westwood

**Affiliations:** 1 Department of Psychology and Neuroscience, Dalhousie University, Halifax, NS, Canada; 2 School of Health and Human Performance, Division of Kinesiology, Dalhousie University, Halifax, NS, Canada

**Keywords:** action sequence, attention, grasping, motor control, sequential movements

## Abstract

Every day we perform consecutive grasping actions to complete our desired goals. Although we have a good understanding about how the brain programs one grasping action towards a single object, we have limited knowledge about how the brain programs a grasping action within the context of a sequential task. The aim of this study was to investigate whether the characteristics of the second object in a sequence of two grasping movements affects how the first object is grasped. In Experiment 1, we explored whether the size and orientation of the second object would affect the grip aperture and grip orientation used to grasp a first cylindrical object that varied between two sizes. Unlike results reported by Hesse and Deubel, the orientation of the second object had no significant influence on the grip orientation used to grasp the first object. However, when the second object was smaller than the first object, participants reached out with a smaller peak grip aperture relative to when the second object was the same size or larger. In Experiment 2 we manipulated object orientation but not size, in a strict replication of Hesse and Deubel’s study. Like Experiment 1, we found no effect of the second object’s orientation on the first grasping movement. Based on these results, it is suggested that the second object within a sequential task will only interfere with the grasping kinematics towards the first object when both action plans have overlapping features, and most importantly this is not dependent on providing a strategic benefit to the overall movement.

## 1 Introduction

Many of our activities of daily living rely on successive actions that require us to reach towards, grasp, lift and transport objects in sequence. Although we know much about the neuroscience of how the brain plans one grasping action towards a single object [as highlighted by reviews from Castiello (2005), Castiello and Begliomini (2008), Grafton (2010), and Turella and Lingnau (2014)], comparatively little is known about the control of grasping in sequential tasks involving multiple objects and actions.

**Figure 1:**
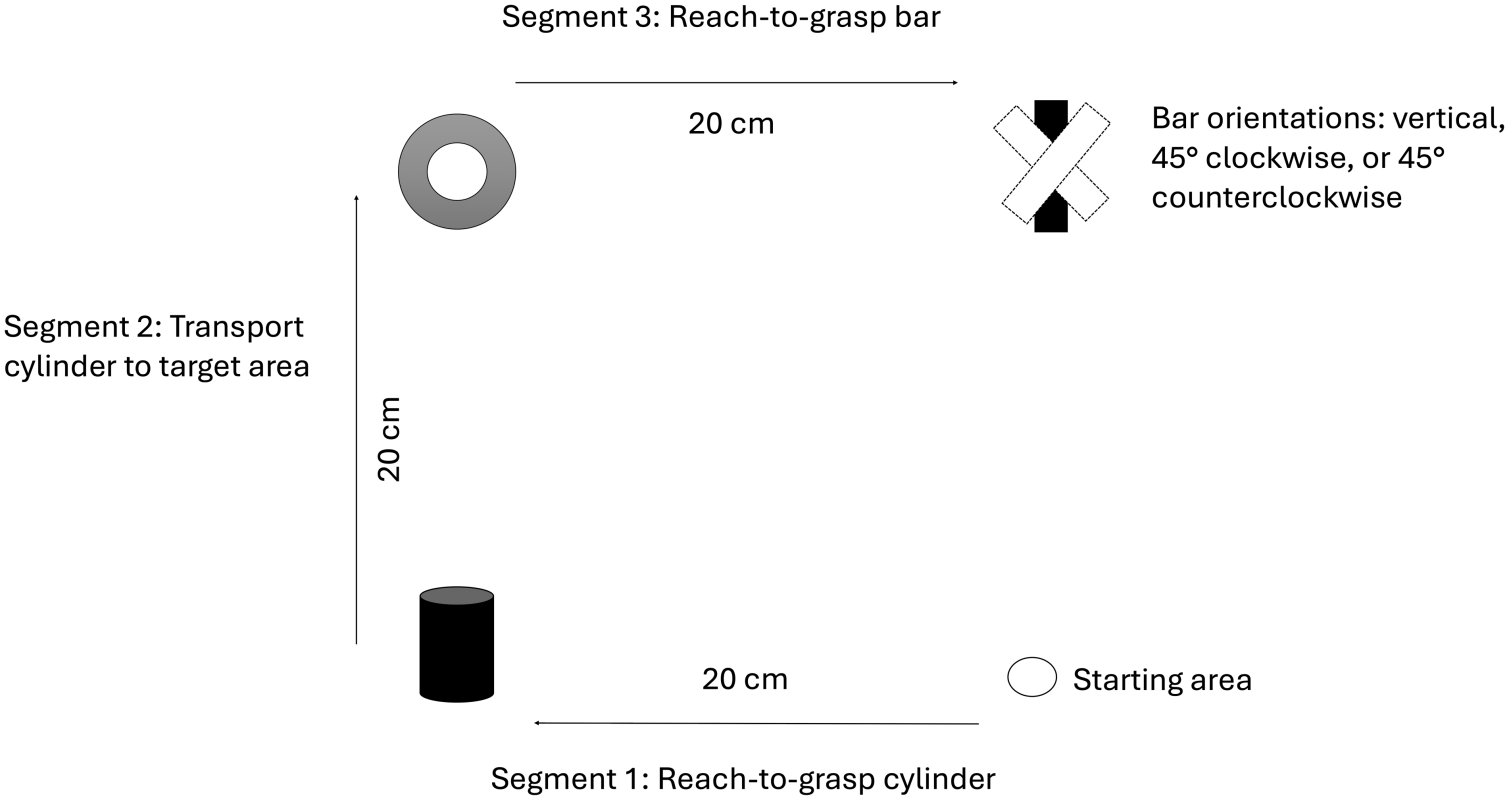
Schematic drawing of the experimental setup used in Hesse and Deubel’s (2010) study. From the starting area, participants would reach to grasp the cylinder (first object), place it within the target area, and then grasp the bar that was either orientated vertically, 45 ° clockwise, or −45 ° counterclockwise (second object).

It has been shown that when participants perform a sequential grasping task attention is not only allocated to the first target but also to the second target of the sequence before the onset of the initial movement (Deubel and Schneider, 2004). Building on this observation, Hesse and Deubel (2010) devised a task in which participants grasped and transported a cylindrical object followed immediately by similar action where the target was a rectangular bar with varying orientations. Participants’ grip orientation for the initial movement was greatly influenced by the orientation of the second object in the grasping sequence despite the fact that the two objects and actions had no functional relationship to each other aside from their sequential order (see Figure 1 for depiction of their experimental set up). The results were interpreted as support for holistic planning when multiple actions are connected in a sequence. Thus, as opposed to treating each action of the sequence in isolation, the whole sequence is programmed together and features of the second action will be implemented within the first action to optimize the movement end-goal (e.g., grip orientation already pre-adjusted to perform the final action as the first action takes place). This explanation converges with the movement integration hypothesis used to explain the one-target advantage when performing sequential reaching actions (Adam et al., 2000, 2001; Bested et al., 2018; Helsen et al., 2001; Khan et al., 2006, 2008, 2010; Mottram et al., 2014; Rand et al., 1997; Rand and Stelmach, 2000) and the end-state comfort effects seen in grasping movements (Rosenbaum et al., 1990, 1993, 1996; Rosenbaum and Jorgensen, 1992).

In contrast to a holistic planning mechanism, which implies a functional integration between sequential movements intended to optimize performance, Hesse and Deubel’s results can also be explained by an interference mechanism in which features of the second action unintentionally influence the motor plan for the first action. This produces unintended blending of multiple competing actions plans, which could either improve, hinder, or have no impact on performance depending on the circumstances. Indeed, it has been shown that properties of objects that are merely distractors can influence the movement kinematics towards a target object (Bonfiglioli and Castiello, 1998; Castiello, 1996; Doyle and Walker, 2001; Howard and Tipper, 1997; Neyedli and Welsh, 2012; Tipper et al., 2002; Sheliga et al., 1994, 1995; Welsh and Elliott, 2004), attention can be simultaneously deployed to all relevant action targets of a sequence (Baldauf et al., 2006; Baldauf et al., 2008), attention towards an object will automatically activate its associated action (Chao and Martin, 2000; Grèzes et al., 2003; Murata et al., 1997; Tunik et al., 2007; Tucker and Ellis, 1998; Valyear et al., 2006), and motor plans for different targets/actions can be processed in parallel (Cisek and Kalaska, 2005, 2010; Chapman et al., 2010; Gallivan et al., 2017; Klaes et al., 2011; Stewart et al., 2013, 2014; Tipper et al., 1998; Wood et al., 2011) and co-optimized (Gallivan et al., 2015, 2016; Kashefi et al., 2024; Meirhaeghe et al., 2023).

To distinguish between these competing models, we adapted Hesse and Deubel’s (2010) study by manipulating two features of the target objects: size and orientation. As argued by Hesse and Deubel, the structure of their task arguably affords a potential efficiency to the actor if the grip orientation used to grasp the initial cylinder is biased towards the orientation of the second rectangular object, such that any potential influence of the second object’s orientation on the first action could reasonably be interpreted as evidence of holistic or integrated planning. However, there is no conceivable mechanical advantage to be gained by incorporating the size of the second object in the sequence into the grip aperture adopted during the reach toward the first object, since the hand must close to the actual size of the first object to grasp and transport it as required in the task. Consequently, any effect of the size of the second object on the grip aperture observed during the movement toward the first object would be more consistent with an interference mechanism rather than a holistic or integrated planning mechanism.

A further caveat about Hesse and Deubel’s (2010) paradigm—and other similar studies (Haggard, 1998; LeBlanc and Westwood, 2016; Seegelke et al., 2012, 2013, 2015)—is that the properties of the first object in the sequence remained the same throughout the experiment. As such, it is possible that the initial action was simply reproduced from memory rather than programmed in real-time using current visual input; prior studies have suggested that such memory-guided actions differ in important ways from visually guided actions (Westwood and Goodale, 2003, 2011; Singhal et al., 2013). It is possible that Hesse and Deubel’s results are unique to actions planned from memory. It is also possible that the effects observed for the second object in the sequence on the initial movement depend on the participant allocating all their attention to that that object, which is possible when the first action is always the same and thus requires little attention.

Experiment 1 was designed to distinguish between holistic planning and interference accounts of interactions between two actions in a sequence, by adapting Hesse and Deubel’s (2010) paradigm with two key differences. First, the size of the first target object was varied across trials to encourage real-time movement planning based on current visual input. Second, both the size and orientation of the second object in the sequence were varied across trials: holistic planning predicts an effect of orientation, but not size, on the first action whereas interference predicts an effect of both size and orientation.

## 2 Experiment 1

### 2.1 Materials and methods

#### 2.1.1 Participants

Nineteen undergraduate students at Dalhousie University participated in the current study in exchange for partial course credit. All were right-handed, had normal or corrected-to-normal vision, and no history of neurological deficit as ascertained by self-report. Each participant provided informed written consent prior to participation in accordance with guidelines established by the Dalhousie University Research Ethics Board.

An Optotrak 3,020 (Northern Digital, Waterloo, ON, CANADA) system was used to record at 200 Hz the three-dimensional locations of IREDs placed on the distal phalanx of the thumb, the lateral surface of the distal phalanx of the index finger, and the styloid process of the radius of the right upper limb. Participants wore liquid–crystal occlusion glasses (PLATO Translucent Technologies, Toronto, ON, Canada) to block visual input during the experiment as indicated in the procedure. A tone was presented via Experiment Builder v1.3 software (SR Research, Osgoode, ON) as the signal for participants to initiate the first action (800 Hz, 250 ms).

#### 2.1.2 Materials

**Figure 2:**
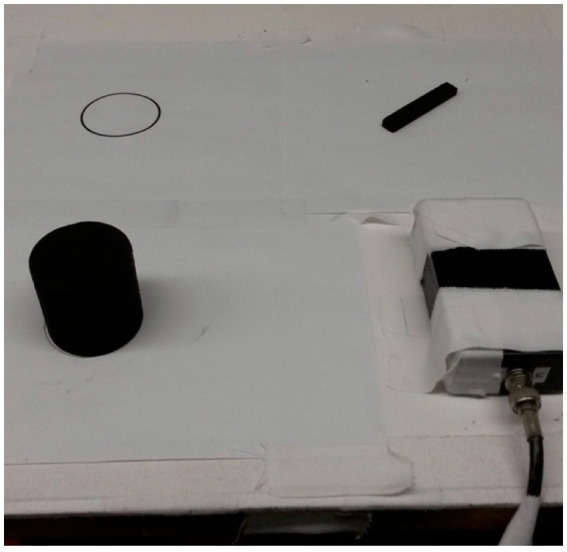
Stimulus layout. This example depicts a trial where the second object is longer than the cylinder’s diameter and is oriented 45° clockwise.

For each trial of the experiment, two black objects were presented simultaneously on a white surface table. The stimulus for the first action of the task was a cylinder located 20 cm to the left of the starting switch. The cylinder was 5 cm tall and did not vary during the experiment, however the diameter took one of two values (5 cm or 6 cm) on a trial-to-trial basis to minimize memory reliance and promote real-time visuomotor control (Westwood and Goodale, 2011). Twenty centimeters above the location of the cylinder was a marked black circle (7 cm diameter). This indicated the target location of where the participants needed to move the cylinder. The stimulus for the second action of the task was a rectangular bar located 20 cm to the right of the target circle. The width and thickness of the bar remained the same throughout the experiment (2 cm x 2 cm). However, the length and orientation of the bar changed on a trial-to-trial basis. Based on the size of the cylinder for a given trial, the bar could either be smaller (3 cm, 4 cm), the same size (5 cm, 6 cm) or bigger (7 cm, 8 cm) and could be orientated vertically, 45 ° clockwise, or −45 ° counterclockwise—see Figure 2 for an example of the stimulus layout.

#### 2.1.3 Procedure

Participants were seated in front of a table during all experimental trials. Each participant performed several practice trials to ensure they understood the requirements of the task. They were first instructed to depress a release button using their pinched right index finger and thumb at the start of each trial. The LCD glasses were opaque at the start of each trial while the experimenter positioned the target objects. Once the objects were correctly positioned the experimenter triggered the start of the trial and the glasses turned transparent to reveal the environment. The start tone was presented 500–1,500 ms later (possible delays were 500, 750, 100, 1,250, or 1,500 ms with equal distribution and randomized trial by trial).

At the sound of the start tone, participants were required to reach, grasp, and pick up (with right thumb and right index finger) the cylinder and to place it in the marked target circle area. Continuing on without interruption, participants were then required to reach, grasp, and pick up the rectangular bar along its long axis using a precision grip, with the index finger and thumb placed at opposite ends of the bar, and transport it to the center of the workspace. Participants were instructed to complete each action as quickly and accurately as possible. The LCD glasses returned to an opaque state 5,000 ms after the initiation tone, such that vision was available during the entire task but occluded at the end of each trial before the stimuli for the next trials were arranged. Each participant performed a total of 180 trials with full randomization: 2 cylinder sizes [5 cm, 6 cm] x 3 relative bar sizes [2 cm smaller, same size, 2 cm larger] x 3 bar orientations [−45° counterclockwise, 0°, 45° clockwise] x 10 repetitions.

#### 2.1.4 Data processing

Offline, a custom Python routine was used to extract movement kinematics from the raw 3D data collected during the experiment. The data was filtered using a second-order dual pass Butterworth filter employing a low-pass cut-off frequency of 12 Hz. Measures extracted from the primary action (the movement to the first object) included peak grip aperture (PGA; the maximum distance between the index finger and thumb achieved during the movement), time to peak grip aperture (TPGA; the time from movement onset to the point of peak grip aperture), reaction time (RT; the time from the onset of the auditory go signal until the velocity of the IRED on the wrist exceeded 30 mm/s for 5 consecutive time samples), movement time (MT; the time from when the wrist IRED exceeded 30 mm/s for 5 consecutive time samples until it dropped below 30 mm/s for 5 consecutive time samples), and grip orientation (the angle of the horizontal projection of the index finger and thumb—0 ° in orientation corresponded to a perfect sagittal line projection, clockwise projections from that line is defined as positive angles and counterclockwise projections as negative angles). Grip orientation was measured at the time of object contact, when the index finger and thumb closed on the object to complete the grasp, consistent with Hesse and Deubel (2010), who reported their effects at the moment of grasp completion. Interactive routines enabled the experimenter to ensure the automated algorithms chose the appropriate values in cases of missing IRED positions. All dependent measures were analyzed within participants, and trials were rejected if any of the measures fell beyond ±3 standard deviations of the individual participants’ mean for that measure (less than 1% of trials were rejected from data analyses).

Each dependent measure was analyzed using a 2 (cylinder size) x 3 (relative rectangular bar size) x 3 (rectangular bar orientation) ANOVA (*α* = 0.05). *Post hoc* analysis was done with Bonferroni-corrected t-tests. Sphericity was evaluated using Mauchly’s test (*α* = 0.05), and Greenhouse–Geisser corrections were applied if needed.

### 2.2 Results

Given the hypotheses under investigation, all analyses focus solely on the kinematic characteristics of the first action in the sequence (i.e., reaching toward and grasping the cylinder) to determine any effects of the features of the next object or action in the sequence. The descriptive statistics for each dependent measure can be found in Table 1.

**Table 1:** Mean (SD) of all dependent measures across first object sizes, presented by the size and orientation of the second object.

Second object (size/orientation)	PGA 6 cm (mm)	PGA 5 cm (mm)	Orientation 6 cm (°)	Orientation 5 cm (°)	RT 6 cm (ms)	RT 5 cm (ms)	MT 6 cm (ms)	MT 5 cm (ms)	tPGA 6 cm (ms)	tPGA 5 cm (ms)
Larger/0°	86.69 (7.9)	81.03 (8.6)	−3.94 (12.3)	−3.66 (13.8)	494 (200)	481 (189)	555 (95)	568 (99)	436 (85)	442 (92)
Larger/−45°	87.42 (8.5)	80.89 (8.2)	−2.36 (13.4)	−5.47 (13.5)	476 (204)	483 (201)	562 (93)	562 (91)	440 (82)	436 (88)
Larger/45°	87.12 (7.4)	81.39 (8.5)	−2.39 (12.9)	−3.22 (13.2)	478 (182)	461 (184)	550 (77)	558 (89)	435 (74)	436 (87)
Same/−45°	86.86 (7.9)	81.83 (8.2)	−3.70 (12.9)	−2.94 (14.2)	475 (193)	470 (175)	561 (84)	553 (83)	444 (81)	436 (78)
Same/0°	87.67 (7.3)	80.45 (8.0)	−2.31 (13.2)	−4.14 (13.1)	460 (200)	494 (196)	555 (88)	563 (82)	433 (82)	443 (78)
Same/45°	87.12 (8.2)	81.31 (7.6)	−3.15 (13.4)	−3.56 (13.0)	487 (166)	485 (199)	573 (96)	557 (82)	443 (86)	431 (81)
Smaller/−45°	86.36 (8.2)	80.58 (8.3)	−3.50 (13.4)	−5.12 (13.2)	480 (192)	477 (183)	558 (88)	568 (89)	440 (86)	439 (88)
Smaller/0°	86.86 (7.8)	80.88 (8.4)	−2.10 (13.1)	−4.42 (13.8)	480 (198)	488 (161)	558 (89)	574 (94)	436 (81)	449 (86)
Smaller/45°	86.16 (8.4)	81.16 (7.9)	−3.23 (12.9)	−3.86 (13.9)	488 (175)	468 (208)	569 (90)	550 (89)	451 (88)	429 (87)

#### 2.2.1 Peak grip aperture

**Figure 3:**
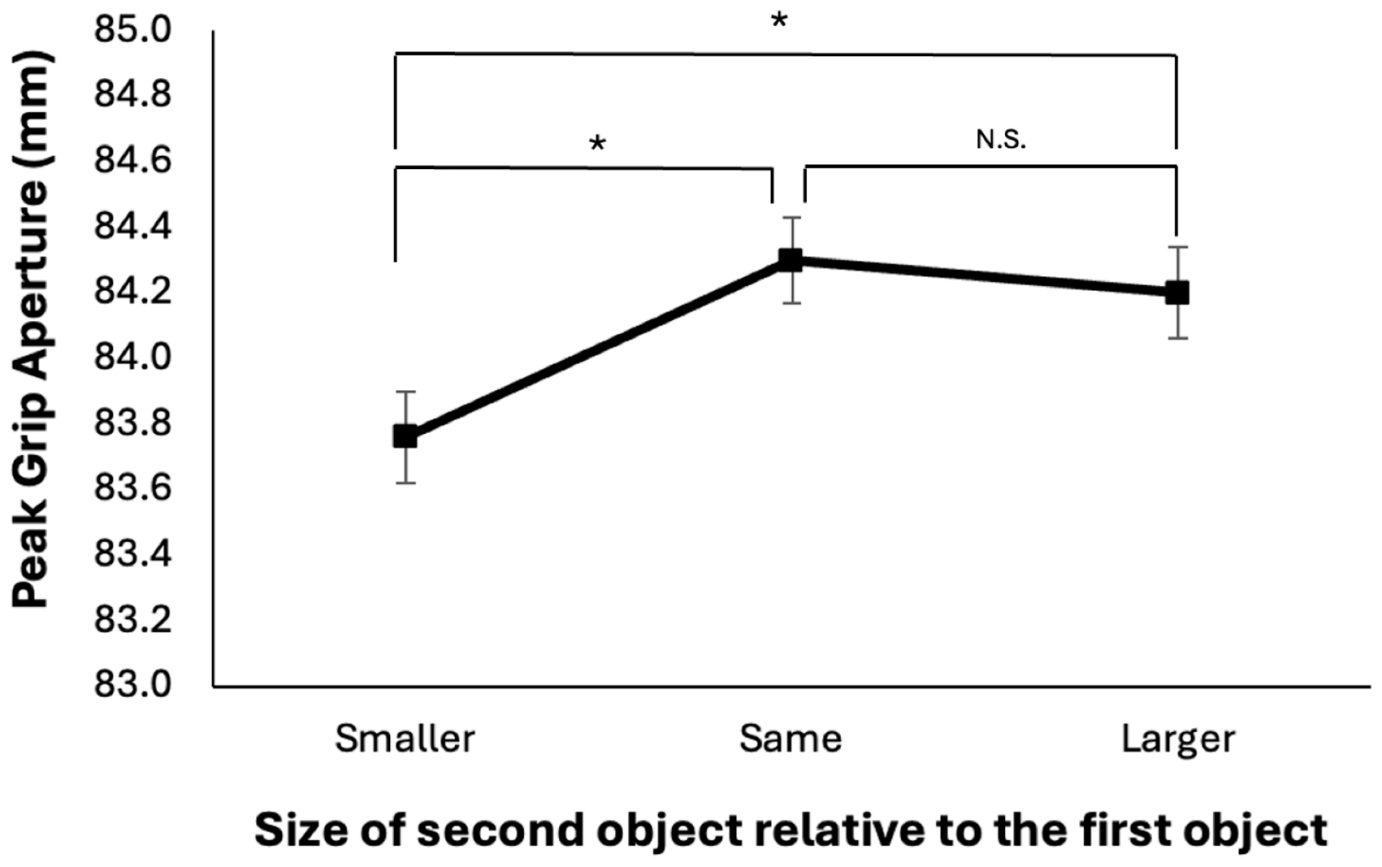
Mean peak grip aperture when performing a grasping action to the first object in relation to the size of the second object. Error bars indicate SEM. “*” denotes a statistically significant difference (*p* < 0.05).

The results revealed no significant interaction between the size of the cylinder’s diameter (first object), size of rectangular object (second object) and orientation of rectangular object (second object), *F*(4, 72) = 1.49, *p* = 0.22, *η_p_^2^* = 0.09. There was also no significant interaction between the size of first object and size of second object, *F*(2, 36) = 0.25, *p* = 0.78, *η_p_^2^* = 0.02; the size of first object and orientation of second object, *F*(2, 36) = 0.68, *p* = 0.52, *η_p_^2^* = 0.04; the size of second object and orientation of second object, *F*(4, 72) = 0.28, *p* = 0.89, *η_p_^2^* = 0.02. As expected, there was a main effect for size of first object, *F*(1, 18) = 406.01, *p* < 0.001, *η_p_^2^* = 0.96. Participants reached out with a larger peak grip aperture for the cylinder that had a diameter of 6 cm (*M* = 87.07 mm, *SE* = 0.11) than the one of 5 cm (*M* = 81.04 mm, *SE* = 0.12). A main effect was also found for the size of the second object, *F*(2, 36) = 6.02, *p* = 0.006, *η_p_^2^* = 0.29. Post-hoc analysis, paired-sample t-test with Bonferroni correction (α = 0.0167), revealed a significant difference between the smaller second object in comparison to the same size object, *t*(18) = −4.59, *p* < 0.01, and the larger size, *t*(18) = −4.34, *p* < 0.01, but no significant difference between the same size and larger size, *t*(18) = 0.38, *p* > 0.01. As seen in Figure 3, participants reached out for the first object with a smaller peak grip aperture when the second object was smaller than the first one (*M* = 83.76 mm, *SE* = 0.14) relative to if the second object was the same size (*M* = 84.32 mm, *SE* = 0.13) or larger (*M* = 84.24 mm, *SE* = 0.14). No main effect of orientation of second object was found, *F*(2, 36) = 0.49, *p* = 0.61, *η_p_^2^* = 0.03.

#### 2.2.2 Grip orientation

**Figure 4:**
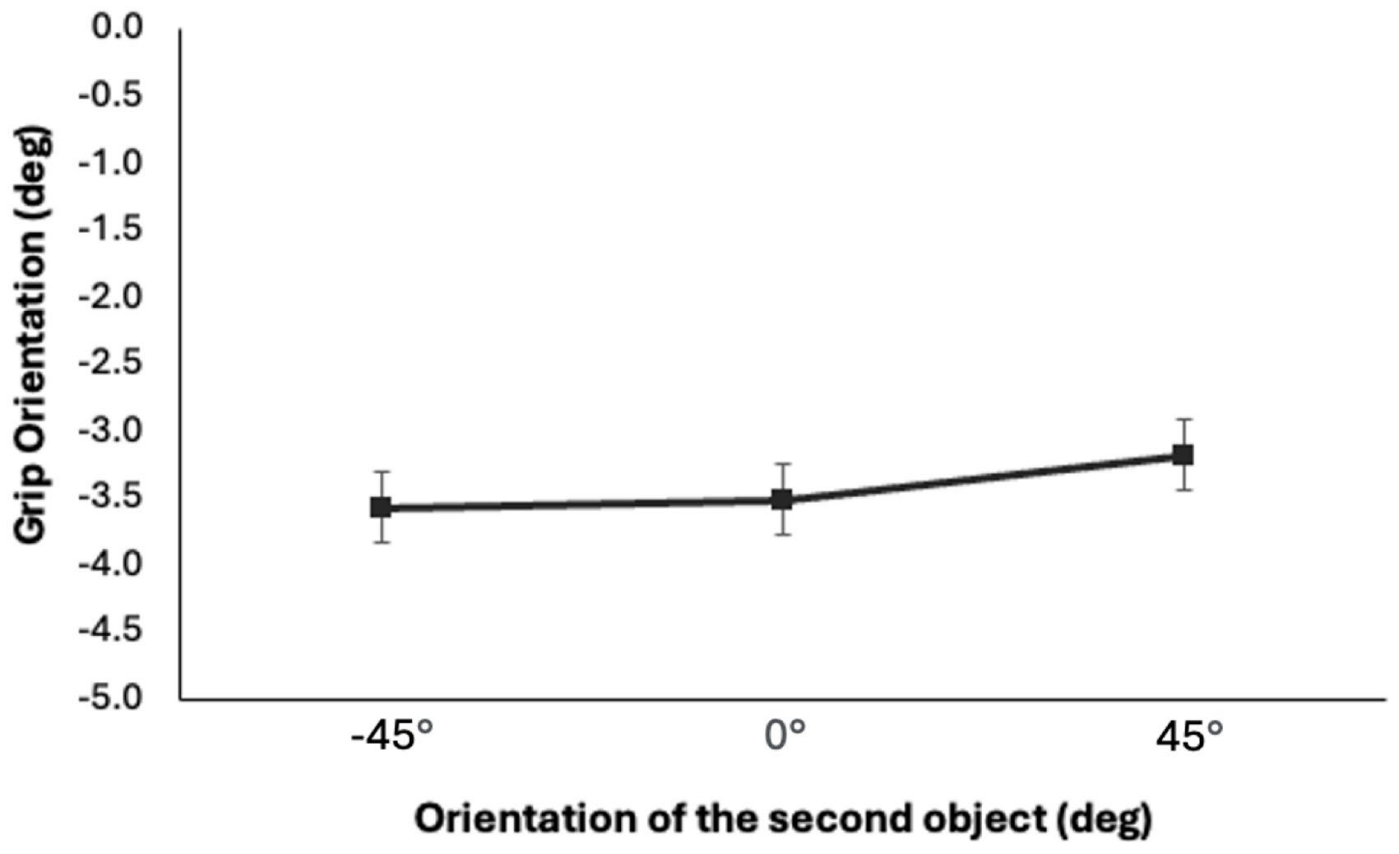
Mean grip orientation when performing a grasping action to the first object in relation to the orientation of the second object. Error bars indicate SEM. No significant differences were found.

No significant interaction was found between size of first object, size of second object and orientation of second object, *F*(4, 72) = 1.15, *p* = 0.34, *η_p_^2^* = 0.07. There was also no significant interaction between size of first object and size of second object, *F*(2, 36) = 0.85, *p* = 0.44, *η_p_^2^* = 0.05, size of first object and orientation of second object, *F*(2, 36) = 0.004, *p* = 0.99, *η_p_^2^* = 0.001 and size of second object and orientation of second object, *F*(4, 72) = 0.88, *p* = 0.48, *η_p_^2^* = 0.05. A main effect of size of the first object was revealed, *F*(1, 18) = 14.6, *p* = 0.002, *η_p_^2^* = 0.49, participants titled their grip orientation more towards the left for the small cylinder (*M* = −4.09 °, *SE* = 0.22) than the large cylinder (*M* = −2.79 °, *SE* = 0.21). No main effect of size of second object, *F*(2, 30) = 0.6, *p* = 0.55, *η_p_^2^* = 0.04 and most importantly no main effect of orientation of second object, *F*(2, 36) = 0.56, *p* = 0.58, *η_p_^2^* = 0.03, as seen on Figure 4.

#### 2.2.3 Reaction time

No significant interaction was found for size of the first object, size of the second object and orientation of second object, *F*(4,72) = 0.63, *p* = 0.64, *η_p_^2^* = 0.04. No significant interaction between size of first object and size second object, *F*(2, 36) = 1.79, *p* = 0.18, *η_p_^2^* = 0.11, size of first object and orientation of second object, *F*(2, 36) = 0.31, *p* = 0.73, *η_p_^2^* = 0.02, and size of second object and orientation of second object, *F*(4, 72) = 0.45, *p* = 0.77, *η_p_^2^* = 0.03. No main effect of size of the first object, *F*(1, 18) = 0.001, *p* = 0.98, *η_p_^2^* = 0.001, size of second object, *F*(2, 36) = 0.02, *p* = 0.98, *η_p_^2^* = 0.001 and orientation of second object, *F*(2, 36) = 0.5, *p* = 0.61, *η_p_^2^* = 0.03.

#### 2.2.4 Movement time

No significant interaction was found for size of the first object, size of the second object and orientation of second object, *F*(4, 72) = 1.72, *p* = 0.16, *η_p_^2^* = 0.10. No significant interaction between size of first object and size of second object, *F*(2, 36) = 2.76, *p* = 0.09, *η_p_^2^* = 0.15, size of first object and orientation of second object, *F*(2, 36) = 3.2, *p* = 0.08, *η_p_^2^* = 0.16, size of second object and orientation of second object, *F*(4, 72) = 1.3, *p* = 0.28, *η_p_^2^* = 0.08. No main effect of size of first object, *F*(1, 18) = 0.02, *p* = 0.89, *η_p_^2^* = 0.001, size of second object *F*(2, 36) = 0.4, *p* = 0.67, *η_p_^2^* = 0.03 and orientation of second object, *F*(2, 36) = 0.35, *p* = 0.71, *η_p_^2^* = 0.02.

#### 2.2.5 Time to peak grip aperture

No significant interaction was found for the size of the first object, size of the second object and orientation of second object, *F*(4, 72) = 1.2, *p* = 0.31, *η_p_^2^* = 0.07. No significant interaction between size of first object and size of second object, *F*(2, 36) = 1.82, *p* = 0.18, *η_p_^2^* = 0.11, no interaction for size of second object and orientation of second object, *F*(2, 36) = 0.41, *p* = 0.80, *η_p_^2^* = 0.03, and no interaction was found for size of first object and orientation of second object, *F*(2, 36) = 1.65, *p* = 0.21, *η_p_^2^* = 0.09. No main effect of size of first object, *F*(1, 18) = 0.39, *p* = 0.54, *η_p_^2^* = 0.54, size of second object, *F*(2, 36) = 0.29, *p* = 0.75, *η_p_^2^* = 0.02 and orientation of second object, *F*(2, 36) = 0.40, *p* = 0.67, *η_p_^2^* = 0.03.

### 2.3 Discussion

The purpose of this study was to gain a better understanding of the planning and execution of sequential grasping actions. More specifically, we attempted to distinguish between two competing accounts of results presented by Hesse and Deubel (2010) that showed effects of the second object in a sequence on the kinematics of the action to the first object. If Hesse and Deubel’s holistic/integrated planning account is correct, we would expect to see effects of the orientation but not size of the second object on grasping kinematics for the first action, but if an interference account is correct then effects of both orientation and size should be observed.

Our results do not clearly support either account. We found a significant effect of the relative size of the second object on peak grip aperture for the first action, but no effect of the orientation of the second object on the grip orientation for the first action. Of course, the latter result is in stark contrast to the robust effect of the second object’s orientation first reported by Hesse and Deubel (2010).

Although our paradigm was modeled on Hesse and Deubel’s (2010) study, several deliberate modifications may explain why we did not replicate their findings. Unlike their study, which used the same size cylinder throughout, we varied the cylinder’s size (6 cm or 5 cm) on a trial-by-trial basis, requiring participants to actively process the first object’s features each time. In addition to varying the orientation (−45°, 0°, or 45°) of the second object, we also varied its size (smaller, same, or larger) to assess whether the first action would be influenced by features that are either strategically beneficial (orientation) and/or irrelevant (size) to the movement sequence.

One likely reason for the discrepancy in our results is that we varied the size of the first object, which may have discouraged reliance on memory-guided control and instead promoted real-time visuomotor processing (Westwood and Goodale, 2003, 2011; Singhal et al., 2013). In contrast, previous studies that found orientation effects used a first object of fixed size (Haggard, 1998; Hesse and Deubel, 2010; Seegelke et al., 2012, 2013, 2015). This methodological difference raises two possibilities: first, that orientation effects are more likely to emerge when the first action is guided by memory; and second, that keeping the first object the same size may allow participants to allocate more attentional resources to planning the second action. Supporting the latter possibility, interference effects from the second object tend to disappear when the initial action demands greater precision (e.g., Hesse and Deubel, 2010, Exp. 2; Rand and Stelmach, 2000).

The presence of a size effect despite these attentional demands instead supports an interference account. This theory posits that even task-irrelevant properties of objects—like the size of the second object—can affect movement kinematics if those features overlap with the planning features of the first action. This is consistent with findings that distractor objects and competing motor plans can influence action selection and execution (e.g., Bonfiglioli and Castiello, 1998; Chapman et al., 2010; Gallivan et al., 2016; Welsh and Elliott, 2004). Partial support for this view was found, as the size of the second object influenced PGA, albeit only when it was smaller.

One explanation for this asymmetry is that shared feature dimensions (size) between the two objects made size more salient than orientation, particularly since the first object had no orientation component. This aligns with theories suggesting that interference arises more strongly when action plans share features (Fournier et al., 2014; Hommel, 2009; Mattson and Fournier, 2008; Stoet and Hommel, 1999). Furthermore, grasping smaller objects may require greater online control (Grol et al., 2007), thereby drawing more attention to the second action and leading to unintentional carryover effects on the initial grasp.

Another possibility, based on the “digit-in-space” theory of grip formation (Smeets et al., 2019), is that aperture tuning for the larger first object overlaps with the required aperture for the smaller second object creating opportunities for interference. However, the smaller first object would reach peak aperture earlier, potentially limiting overlap with the larger second object and explaining the lack of an effect in that direction. Future work could test this by manipulating the starting hand posture (e.g., starting with fingers apart), which might reverse the size effect.

Our findings challenge the holistic planning hypothesis and raise the possibility that Hesse and Deubel’s (2010) orientation effects were driven by the constancy of the first object, which may have enabled memory-based rather than real-time visuomotor control. A direct replication using a fixed first object could clarify whether orientation effects emerge only under reduced visuomotor demands. Conversely, a failure to replicate would further undermine the holistic account and reinforce the view that interference is constrained to features shared across consecutive movements.

## 3 Experiment 2: replication of Hesse and Deubel’s study

The purpose of this second experiment was to perform a strict replication of Hesse and Deubel’s (2010) study to clarify the results from Experiment 1. We directly contacted the main author (C. Hesse), and they provided us with some supplementary materials associated to their study (more detailed explanations about their methodology and a video showing a participant performing a sample trial). This allowed us to directly mimic as close as possible their exact paradigm and experimental setup.

### 3.1 Materials and methods

#### 3.1.1 Participants

Twenty undergraduate students at Dalhousie University participated in the current study in exchange for partial course credit. All were right-handed, had normal to corrected-to-normal vision, and no history of neurological deficit as ascertained by self-report. Each participant provided informed written consent prior to participation in accordance with guidelines established by the Dalhousie University Research Ethics Board.

#### 3.1.2 Materials

For each trial of the experiment, two wooden objects were presented simultaneously on a white surface table. The stimulus for the first action was a red cylinder located 20 cm to the left of the starting switch. The cylinder measured 5.5 cm tall and 4 cm in diameter and the same cylinder was used for all trials. Twenty centimeters above the location of the cylinder was a marked circled area colored in either red or yellow. The red circle was 4.5 cm in diameter and the yellow circle was 6 cm. This indicated the target location of where the participants needed to move the cylinder. The stimulus for the second action was a black rectangle bar measuring 5 cm in length and 2 cm in thickness and located 20 cm to the right of the target circle. The same bar was used throughout the experiment. However, on a trial-by-trial basis the bar could be oriented vertically (0°), 45 ° clockwise, or—45 ° counterclockwise.

An Optotrak 3,020 (Northern Digital, Waterloo, ON, CANADA) system was used to record at 200 Hz the three-dimensional locations of IREDs placed on the distal phalanx of the thumb, the lateral surface of the distal phalanx of the index finger, and the styloid process of the radius of the right upper limb. Participants wore liquid–crystal occlusion glasses (PLATO Translucent Technologies, Toronto, ON, Canada) to block visual input during the experiment as indicated in the procedure. A tone was presented via Experiment Builder v1.3 software (SR Research, Osgoode, ON) as the signal for participants to initiate the first action (800 Hz; 250 ms). A chin rest was also used and placed at the edge of the table in front of the first object to maintain a constant head position throughout the experiment. It is important to note that we did not use a chin rest in our first experiment.

#### 3.1.3 Procedure

Participants were seated comfortably in front of a table during all experimental trials with their head placed upon a chin rest in a well-lit room. Each participant performed six practice trials to ensure they understood the requirements of the task. They were first instructed to depress a release button using their pinched right index finger and thumb at the start of each trial. The LCD glasses were opaque at the start of each trial while the experimenter positioned the target objects. Once the objects were correctly positioned the experimenter triggered the start of the trial and the glasses turned transparent to reveal the environment. The start tone was presented 1,000 ms after the preview period.

At the sound of the start tone, participants were required to reach, grasp, and pick up (with right thumb and right index finger) the cylinder and to place it in the marked target circle area. The target circle was either the red circle (difficult condition) or the yellow circle (easy condition). However, the target circle was presented in blocks of trials, and it was counterbalanced among participants in terms of whether they performed the first block of trials under the ‘difficult condition’ or the ‘easy condition’ first. Once the first object was placed within the circle area, participants were then required to reach, grasp, and pick up the rectangular bar along the front-to-back axis and place it in the center of the workspace (no specified location).

Participants were instructed to complete each action as quickly and accurately as possible. The LCD glasses returned to an opaque state 4,000 ms after the initiation tone, such that vision was available during the entire task but occluded at the end of each trial before the stimuli for the next trials were arranged. Each participant performed a total of 60 trials (10 trials for each type of orientation performed under the two different blocks).

#### 3.1.4 Data processing

Data processing and exclusion procedures were identical to those described in Section 2.1.4 for Experiment 1, with the exception that peak grip aperture and time to peak grip aperture were not analysed for this experiment.

Each dependent measure was analyzed using a 3 (orientation of bar) x 2 (placing difficulty) repeated-measures ANOVA (*α* = 0.05). Sphericity was evaluated using Mauchly’s test (*α* = 0.05), and Greenhouse–Geisser corrections were applied if needed.

### 3.2 Results

The descriptive statistics for each dependent measure can be found in Table 2.

**Table 2:** Mean (SD) of all dependent measures when grasping a cylinder (first object) in relation to the orientation of the second object for each type of condition.

Orientation of second object (deg)	Grip orientation (°)–easy	Grip orientation (°)–difficult	RT (ms)–easy	RT (ms)–difficult	MT (ms)–easy	MT (ms)–difficult
−45°	−3.63 (8.1)	−3.97 (8.2)	351 (72)	359 (100)	517 (71)	526 (87)
0°	−3.32 (8.2)	−2.93 (8.0)	340 (89)	357 (94)	513 (77)	527 (74)
45°	−3.88 (8.9)	−3.05 (8.1)	347 (78)	356 (79)	517 (68)	538 (88)

#### 3.2.1 Grip orientation

**Figure 5:**
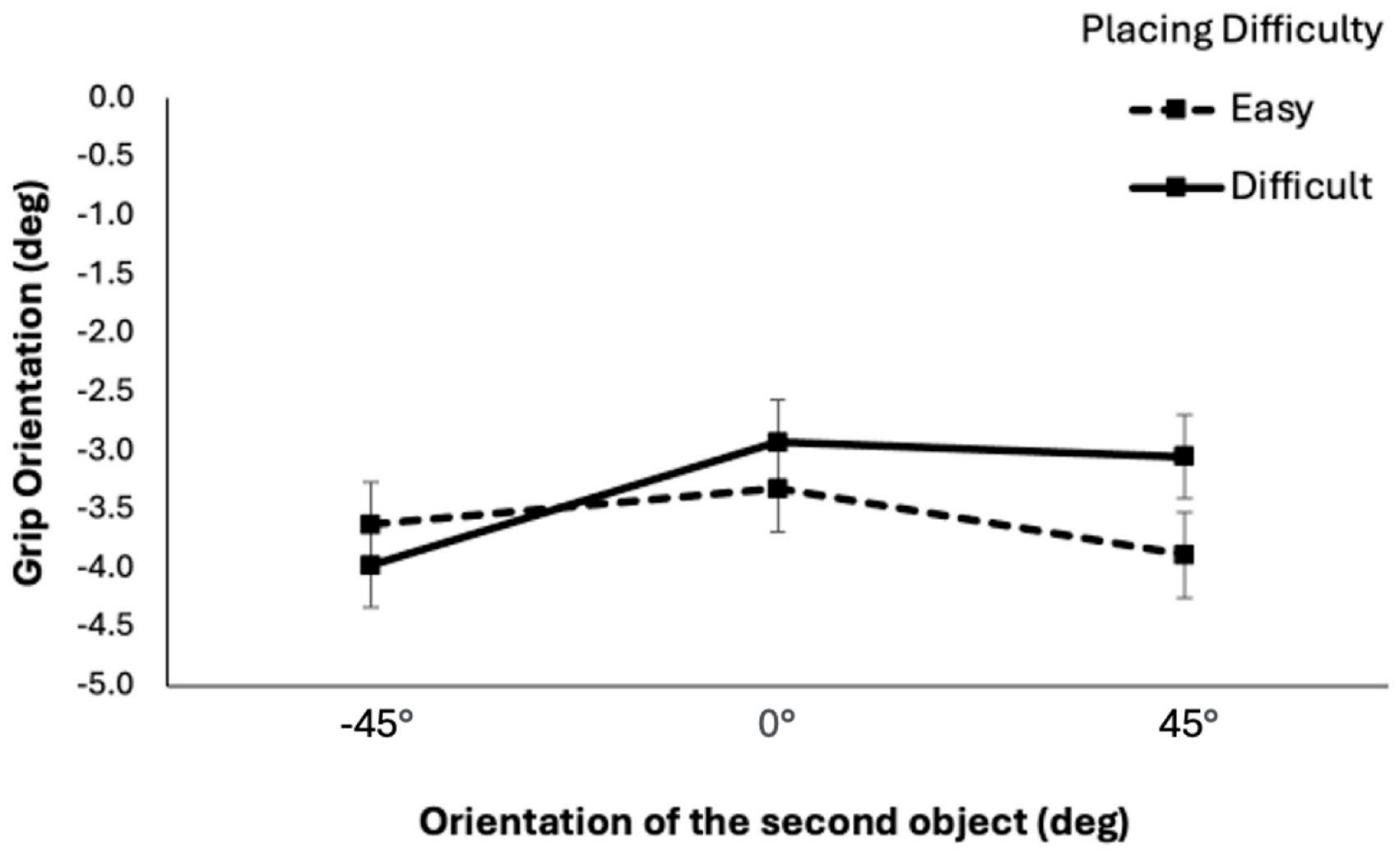
Mean grip orientation when performing a grasping action to the first object in relation to the orientation of the second object. The easy task (bigger placing area) is depicted with the dashed lines and difficult task (smaller placing area) is depicted in with the solid black line. Error bars indicate SEM. No significant differences were found.

No significant interaction was found between the orientation of the bar (second object) and the placing difficulty, *F*(2, 38) = 1.07, *p* = 0.35, *η_p_^2^* = 0.05. There was no main effect of placing difficulty, *F*(1, 19) = 0.08, *p* = 0.78, *η_p_^2^* = 0.005, and no main effect of orientation of the second object, *F*(2, 38) = 1.19, *p* = 0.32, *η_p_^2^* = 0.06, as seen in Figure 5.

#### 3.2.2 Reaction time

There was no significant interaction between the orientation of the second object and the placing difficulty, *F*(2, 38) = 0.36, *p* = 0.69, *η_p_^2^* = 0.02. There was no main effect of orientation of the second object, *F*(2, 38) = 0.49, *p* = 0.62, *η_p_^2^* = 0.03, and no main effect of placing difficulty, *F*(1, 19) = 1.83, *p* = 0.19, *η_p_^2^* = 0.09.

#### 3.2.3 Movement time

There was no significant interaction between the orientation of the second object and the placing difficulty, *F*(2, 38) = 0.12, *p* = 0.89, *η_p_^2^* = 0.01. There was no main effect of orientation of the second object, *F*(2, 38) = 0.11, *p* = 0.82, *η_p_^2^* = 0.01, and no main effect of placing difficulty, *F*(1, 19) = 1.3, *p* = 0.26, *η_p_^2^* = 0.07.

### 3.3 Discussion

The aim of this study was to replicate Hesse and Deubel’s (2010) findings to clarify the results from Experiment 1. Despite strictly matching their methodology, we failed to replicate their reported effects. Consistent with Experiment 1, no influence of the second object’s orientation on the grip orientation for the first object was observed. Additionally, we found no significant differences in movement time (MT) or reaction time (RT) as a function of placing task difficulty.

Notably, the difference in index of difficulty (ID; Fitts, 1954) between the easy and difficult tasks was only 0.3 bits—far below the threshold typically associated with reliable MT differences (≥1 bit; Rand et al., 1997; Rand and Stelmach, 2000). This small difference likely accounts for the null MT effect and further suggests that Hesse and Deubel’s findings may have been atypical. As for RTs, their participants initiated actions approximately 100 ms faster than those in the present study (see Table 3), which may explain the discrepancy. However, this does not support holistic planning. If anything, the longer RTs in our study may have allowed participants sufficient time to inhibit automatic motor activation from the second object, consistent with an interference account rather than strategic integration of future movement parameters.

**Table 3:** Comparing the average (SE) reaction time for the easy and difficult placing task from Hesse and Deubel’s study and our current study.

Reaction time	Hesse and Deubel (2010)	Current study
Easy task	242 ms (13)	346 ms (17)
Difficult task	260 ms (12)	357 ms (20)

This remains speculative, as both studies instructed participants to initiate and perform movements as quickly and accurately as possible, with no time constraints on movement initiation. However, only two of our twenty participants exhibited RTs within the faster range reported by Hesse and Deubel (2010), leaving the discrepancy unexplained. One possible factor is sample composition: their participants included graduate students, while ours were predominantly first-year undergraduates. This difference in prior exposure to experimental tasks may have contributed to slower RTs in our sample due to reduced familiarity with task demands or stimulus–response timing.

Another methodological difference was the motion capture system used for the study. Hesse and Deubel used an electromagnetic system with bulkier, tethered sensors, whereas we used a less intrusive optical system. It is possible that the more natural, unconstrained movements in our setup reduced conscious monitoring of the grasping action. Prior research suggests that unnatural or consciously monitored movements are more susceptible to perceptual interference due to increased dorsal–ventral stream interaction (Navon and Ganel, 2020). Thus, the physical constraints in their setup may have heightened ventral stream involvement, making participants more vulnerable to interference from the second object’s orientation.

Participants in their study received monetary compensation, whereas ours received partial course credit (e.g., bonus points). While the impact of this difference on the results is unclear, monetary rewards are generally considered more motivating and may have led to greater participant engagement, potentially influencing processing during the trials. Research shows that stimuli linked to higher rewards capture attention more effectively (Anderson, 2016; Chelazzi et al., 2013; Failing and Theeuwes, 2017), and can affect movement trajectories (Chapman et al., 2014; Moher et al., 2015) and endpoints (Neyedli and LeBlanc, 2017). However, these effects are typically observed within the same experiment comparing high- and low-reward stimuli, rather than across different studies.

Regardless of the factors contributing to the discrepancies between our findings and those of Hesse and Deubel (2010), we can now conclude that, within the context of our experimental setup, the orientation of the second object does not influence the initial grasping action. This holds true even when the first object remains constant throughout the experiment and in the absence of any variation in the size of the second object.

It is also important to note that two studies with relatively small sample sizes may be insufficient to reliably estimate an actual effect, and replication uncertainty may account for the discrepancies observed (Hedges and Schauer, 2019). More experiments of a similar nature should be conducted to further clarify the evidence for the existence (or lack) of an effect.

## 4 General discussion

The main goal was to further refine our current understanding about how sequential grasping actions are programmed and executed. We wanted to explore whether the kinematics (e.g., PGA and grip orientation) of the first grasping action of a sequence would be affected by the features of the second object/action of the sequence and more specifically what specific characteristics of the second object (e.g., orientation vs. size) can influence how the first grasping action is executed and why it happens (e.g., action efficiency or interference based on parallel processing).

In our first study we showed that when participants grasped the first object (e.g., cylinder) their grasping kinematics were not influenced by how the second object (e.g., rectangular bar) was oriented. However, it was shown that when the second object was smaller than the diameter of the cylinder, participants PGA was significantly smaller when grasping the cylinder relative to when the second object was larger or the same size.

Since these results were not consistent with previous results reported by Hesse and Deubel (2010), we conducted a replication of their study and failed to produce the same results. Our failure to replicate the orientation effect from Hesse and Deubel directly challenges the premise of the holistic planning theory: a process which features of future actions are incorporated into the control of the present movements to optimize performance across an action sequence. As a result, this prompts a reframing of the current experiment as an initial test of whether—and how—upcoming actions can influence ongoing motor performance. This change in perspective shifts the emphasis from confirming a theory to clarifying the current empirical findings.

While grip orientation was unaffected by the future object’s orientation, the influence of its size—despite being irrelevant to the initial action’s kinematics—suggests that size features of future targets can indeed blend into earlier movements. This supports an interference-based account over a strategic one. If participants had been strategically incorporating features of the second object to optimize the sequence, we would have expected the orientation—an actionable feature with a potential payoff in second-step efficiency- to show an effect, not the non-beneficial size feature.

One plausible explanation as to why size but not orientation has an influence on the first action is that size is a shared feature across both actions/objects within our sequential task paradigm. Specifically, the first object (a cylinder) varied in size but had no salient orientation. In contrast, the rectangular second object varied in both size and orientation. The absence of an orientation to compare against in the first object may have nullified any influence of the second object’s orientation. This supports the idea that interference effects are feature-specific and depend on overlap across representational domains (Fournier et al., 2014; Hommel, 2009). Specifically, the planning for size becomes susceptible to interference since both upcoming and current actions require similar dimensional processing, whereas this is not the case with the orientation feature.

The key finding from Experiment 1 was that grip aperture for the first action was influenced by the size of the second object, but only when it was smaller. This asymmetric effect suggests that interference arises when features of the second action share dimensions with and potentially compete for resources involved in the first action (Cisek, 2007). A smaller second object may demand more precise visuomotor control (Grol et al., 2007), drawing attentional resources and inadvertently biasing the initial grip to accommodate the upcoming precision demand. In contrast, a larger second object does not introduce the same precision requirements, nor does it share overlapping constraints that would necessitate modification of the first action. Specifically, since participants were starting with their thumb and index finger pinched together, the trajectories of the fingers must move through the PGA required for the smaller second object as they tune their aperture towards the larger first object. Whereas this does not happen in the context of a larger second object since PGA for the first smaller object will be attained without needing to move through the PGA required for the larger object. Since there is no overlap, the second action can presumably be successfully inhibited, and there is no interference.

Another possibility, related to an interference-based account, is that participants may automatically engage in visual comparisons between objects that are simultaneously present in the workspace. Specifically, size may trigger such comparisons more readily than orientation, either because size is inherently more visually salient or because our experimental setup encouraged it; both objects varied in size, but only the second object varied in orientation. This raises the possibility that orientation-based interference did not occur simply because the first object (a cylinder) lacked an inherent orientation, leaving nothing for participants to visually align with or compare. Thus, the absence of a shared orientation feature may have precluded any interference or blending of that dimension into the first action.

The absence of an orientation effect alongside the presence of a size-based interference effect suggests that sequential motor planning is not automatically governed by a holistic planning process. Rather, our findings point toward a feature-specific interference process, in which overlap in perceptual and/or motor dimensions, such as size, can lead to unintentional blending across action plans. This may reflect competition for shared visuomotor resources or an automatic coupling between similar motor parameters, rather than a top-down strategy aimed at optimizing the action sequence. One additional possibility is that visual size comparisons between concurrently visible objects occur more readily than orientation comparisons, especially in cases where the first object lacks a discernible orientation, as was true in our design. Without a meaningful reference for alignment, orientation information from the second object may be effectively filtered out. Future work should manipulate the orientation properties of both objects or increase the functional relevance of orientation in the first action to assess whether such effects are context-dependent or simply absent when engaging real-time visuomotor control.

An additional consideration is that this context-dependence may extend beyond object features to include the broader spatial layout of the task. In the present design, the endpoint of the first action was spatially independent from the second object, which may have reduced the relevance of incorporating information about the upcoming action into the initial grasp. Therefore, it is possible that more closely linked spatial arrangements between sequential targets could increase the likelihood that features of the second object, including orientation, are incorporated into the first action, particularly when this would support smoother transitions between movements (i.e., movement optimization). Under a holistic planning account, such effects would be taken to reflect the pre-planning of the entire action sequence, with spatial constraints shaping how multiple movement parameters are jointly specified from the outset. However, if these effects only emerge when certain spatial relationships increase overlap between otherwise independent action plans, they may still be more simply explained within an interference/competition framework, where the spatial layout of the task influences the degree of interaction between concurrent motor plans rather than reflecting true sequence-level integration.

In conclusion, instead of validating the assumption of a holistic planning framework, our findings indicate a need to reconsider the planning of sequential actions as a process in which shared features, whether beneficial or not, can unintentionally blend across temporal boundaries and shape the kinematics of ongoing actions as they unfold in real time.

## Data Availability

The original contributions presented in the study are included in the article/supplementary material, further inquiries can be directed to the corresponding author.
